# Chemotherapy is associated with increased survival from colorectal signet ring cell carcinoma with distant metastasis: A Surveillance, Epidemiology, and End Results database analysis

**DOI:** 10.1002/cam4.2054

**Published:** 2019-03-12

**Authors:** Tao Shi, Mengxi Huang, Dong Han, Xinyi Tang, Yanyan Chen, Zhiping Li, Chao Liu, Dan Xiang, Ting Wang, Yitian Chen, Rui Wang, Zengjie Lei, Xiaoyuan Chu

**Affiliations:** ^1^ Department of Medical Oncology Jinling Hospital, Medical School of Nanjing University Nanjing, Jiangsu Province People’s Republic of China; ^2^ Department of Medical Oncology Jinling Hospital, Nanjing Clinical School of Southern Medical University Nanjing, Jiangsu Province People’s Republic of China; ^3^ Department of Medical Oncology Jinling Hospital, Nanjing Clinical School of Nanjing Medical University Nanjing, Jiangsu Province People’s Republic of China

**Keywords:** chemotherapy, peritoneum metastasis, SEER database, signet ring cell carcinoma, surgery

## Abstract

**Background:**

Colorectal signet ring cell carcinoma (SRCC) is a rare histological subtype of colorectal adenocarcinoma with high metastatic frequency compared to non‐SRCC colorectal cancer (NOS). The aim of this study was to analyze prognostic factors of colorectal SRCC with different metastatic sites and evaluate impacts of various therapies for metastatic colorectal SRCC.

**Methods:**

Patients with NOS and SRCC were from the Surveillance, Epidemiology, and End Results (SEER) database during 2010‐2014. χ^2^ tests were used to compare data significance. Kaplan‐Meier and COX models were used to analyze the differences in the survival. Propensity‐matched analyses were used to adjust numerical differences.

**Results:**

Among the 173 460 patients, 1932 (1.11%) patients had colorectal SRCC. In univariate analysis, older age, male sex, and peritoneum metastasis were associated with higher mortality risk. The peritoneum was both the site with the highest metastatic frequency and the site with the worst prognosis in SRCC. In the COX regression model, peritoneum‐metastatic SRCC patients receiving chemotherapy had better survival than patients treated with surgery.

**Conclusions:**

Our study analyzed the unique metastatic pattern of colorectal SRCC toward different sites and found that compared to surgery, chemotherapy was associated with better survival for colorectal SRCC patients with distant metastasis, which provided insights for future SRCC patient treatment.

**Highlights**

- The peritoneum was the site with the highest metastatic frequency and the worst prognosis for colorectal signet ring cell carcinoma (SRCC) patients. Compared to nonmetastatic colorectal SRCC patients, chemotherapy was associated with better survival for colorectal SRCC patients with distant metastasis, providing insights for future SRCC patient treatment.

## 1 INTRODUCTION

Colorectal cancer (CRC) remains the second leading cause of mortality related to cancer in the United States^1^ and is a major health burden worldwide. Among various types of CRCs, colorectal signet ring cell carcinoma (SRCC) has received much attention in recent years. SRCC was first described in 1951 by Laufman and Saphir.^2^ As reported in many studies, SRCC mostly originates from the undifferentiated stem cells of colorectal mucosa, so SRCC often presents poor differentiation, diffuse infiltration, rapid growth, and high metastatic frequency.^3^, ^4^ Several studies have reported the molecular and genetic patterns of SRCC, which contribute to its high metastatic frequency, and partially explain the poor prognostic outcome of metastatic SRCC patients.^6^, ^7^

Further studies have found that many independent prognostic factors are associated with poor survival of SRCC, including age, sex, tumor size, tumor grade, and primary site.^8^, ^9^ In addition, the treatment of colorectal SRCC patients has rapidly improved in recent years. Currently, surgical resection remains the first consideration for the management of colorectal SRCC patients; however, the use of other therapeutic combinations has increased.^10^ Hugen et al^5^ evaluated the efficacy of adjuvant chemotherapy in colorectal SRCC and suggested that stage II and stage III SRCC patients could benefit from adjuvant chemotherapy. Also, in 2017 studies showed that preoperative radiotherapy improved survival of locally advanced colorectal SRCC patients.^9^, ^11^ However, more importantly, due to the relatively high metastatic frequency of SRCC, site‐specific metastasis of colorectal SRCC may have different impacts on survival outcome.^12^ Few studies have focused on the prognostic differences related to different metastatic sites, which hinders the survival improvement of site‐specific colorectal SRCC patients.

To the best of our knowledge, this is the first systematic retrospective study focusing on survival and treatment of site‐specific colorectal SRCC, with a large sample of SRCC patients from the Surveillance, Epidemiology, and End Results (SEER) database. We analyzed the occurrence frequency and prognostic factors of colorectal SRCC and further analyzed the surgery, radiotherapy, and chemotherapy impacts on specific metastatic sites.

## 2 MATERIALS AND METHODS

### 2.1 Data source

The SEER program provides information on cancer incidence and mortality in the USA, consisting of 18 cancer registries and covering approximately 28% of the US population (http://www.seer.cancer.gov). SEER is supported by the Surveillance Research Program (SRP) in NCI's Division of Cancer Control and Population Sciences (DCCPS). Data for this research were obtained from the SEER database submitted in November 2017, containing patient data from 1973 to 2015.^13^

### 2.2 Patient selection

**Figure 1:**
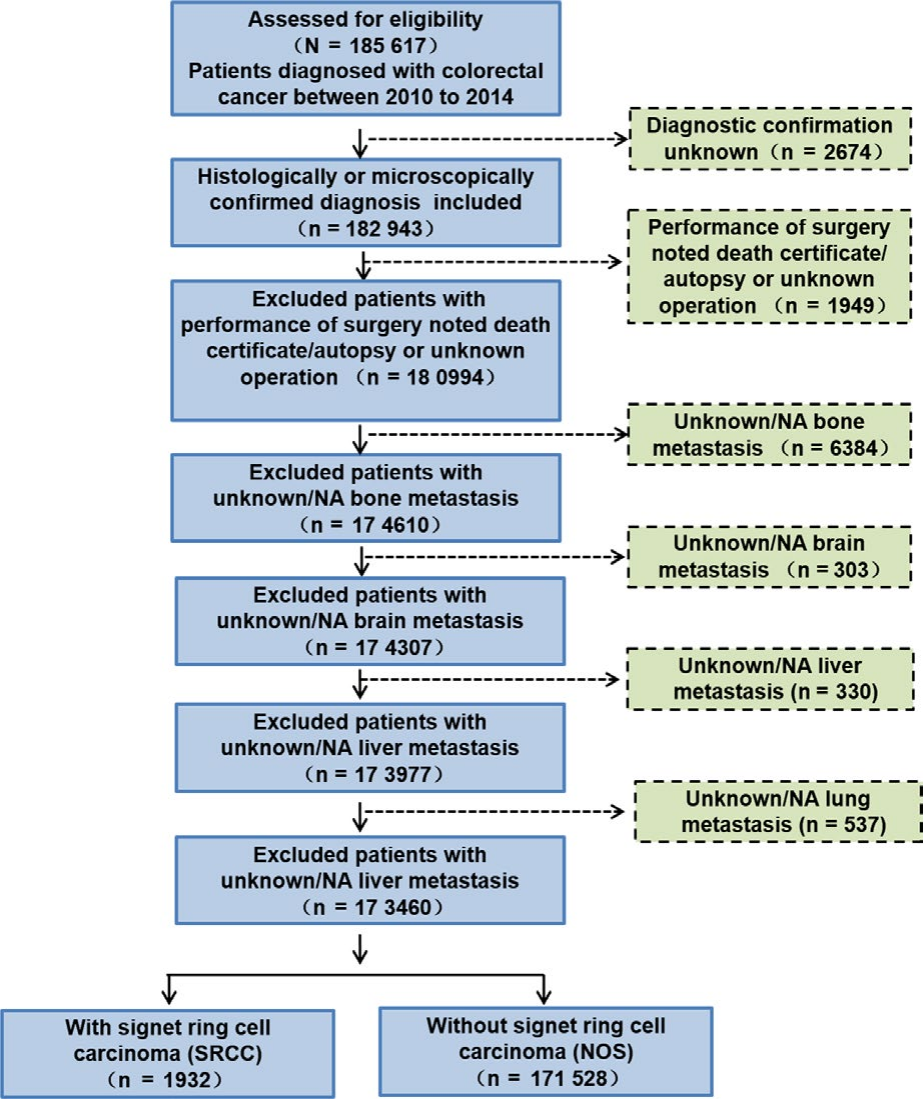
Flowchart for creation of the Surveillance, Epidemiology, and End Results (SEER) patient dataset

The patients for our research were collected from the SEER database for 2010‐2014 that was diagnosed as colorectal cancer (CRC), considering that the site‐specific metastasis data were available since 2010; and 185 617 colorectal patients were assessed for eligibility. We excluded patients with unknown diagnostic confirmation (n = 2674), patients with performance of surgery, noted death certificate/autopsy or unknown operation (n = 1949), patients with unknown/NA bone metastasis (n = 6384), patients with unknown/NA brain metastasis (n = 303), patients with unknown/NA liver metastasis (n = 330), and patients with unknown/NA lung metastasis (n = 537). Finally, 173 460 patients were included for analysis, with 1932 SRCC patients (ICD‐O‐3, 8490) and 171 528 non‐signet ring cell carcinoma (NOS) patients (Figure 1).

### 2.3 Statistical analysis

SEER*Stat software (version 8.3.5) was used to calculate age‐standardized incidence rates (IRs) and incidence rate ratios (IRRs) with 95% confidence intervals (CIs), and R (version 3.5.0) was used to analyze patient records downloaded from the SEER database. χ^2^ tests were used to compare numbers of SRCC and NOS patients with various prognostic factors including age, sex, race, tumor grade, AJCC stage, T/N/M stage, metastatic sites, surgery, chemotherapy, and radiotherapy.

The Kaplan‐Meier method with log‐rank test was used to compare OS (overall survival) and CSS (cause‐specific survival) among groups with different cancer types or different metastatic sites. The Cox proportional hazard regression model with hazard ratio (HR) and 95% CI was used to analyze prognostic factors for survival outcomes in SRCC and NOS patients. Variables with *P* < 0.05 in the univariate analysis were selected into multivariate analyses. Prognostic factors in univariate analyses or multivariate analyses with *P* < 0.05 were considered as statistically significant. Forest plots were used to compare the impact of treatment strategies among different SRCC metastatic subgroups.

The propensity score matching (PSM) was performed to reduce possible bias to a minimum in this study because the number of patients with SRCC and NOS was quite different. The variables matched included in the regression were age, sex, and race. We used χ^2^ tests to examine the covariates balance between the two groups (SRCC and NOS). The survival comparisons later performed for PSM patients used the same methods as those in the primary analysis. The datasets analyzed and computer code used are available from the corresponding author upon request.

## 3 RESULTS

### 3.1 Patient characteristics

A total of 171 528 CRC patients with SRCC/NOS were collected from the SEER database during the 5‐year research period from 2010 to 2014. Among these patients, 1932 were SRCC patients and the other 171 528 were NOS patients (Table 1). In this cohort, patients older than 65 years comprised the majority in both NOS (56.48%) and SRCC (51.76%) subtypes. The *P* value of sex was >0.05, indicating that sex may not be an independent factor between NOS and SRCC. Also, compared to NOS patients, SRCC patients presented more poorly differentiated tumor grade (64.91% vs 13.21%), more advanced AJCC stage (76.04% vs 44.05% in III, IV), more advanced T stage (78.73% vs 56.58% in T3/T4), and more advanced N stage (60.82% vs 35.18% in N1/N2). Importantly, regarding M stage, SRCC patients were more likely to have metastasis than NOS patients (39.13% vs 19.08% in M1), so that it was necessary to analyze the impact of SRCC with site‐specific metastasis. Lastly, there was no large difference in NOS and SRCC patients treated with surgery (84.47% vs 78.78%) or radiotherapy (10.33% vs 7.19%), but more SRCC patients received chemotherapy than NOS patients (54.81% vs 36.65%). Other detailed clinicopathological characteristics between NOS and SRCC patients are also presented in Table 1.

**Table 1:** Clinicopathological characteristics of NOS and SRCC patients and of NOS and SRCC patients after propensity score matching (PSM)

Variable	NOS n = 171 528 (%)	SRCC n = 1932 (%)	*P*	NOS n = 1932 (%) (PSM)	SRCC n = 1932(%) (PSM)		*P*
Age (y)							
<65	74657 (43.52)	932 (48.24)		932 (48.24)	932 (48.24)		
≥65	96871 (56.48)	1000 (51.76)	<0.001	1000 (51.76)	1000 (51.76)	1	
Gender							
Male	89264 (52.04)	997 (51.6)		997 (51.6)	997 (51.6)		
Female	82264 (47.96)	935 (48.4)	0.720	935 (48.4)	935 (48.4)	1	
Race							
White	133717 (77.96)	1584 (81.99)		1584 (81.99)	1584 (81.99)		
Black	21055 (12.27)	201 (10.4)		201 (10.4)	201 (10.4)		
Other/Unknown	16756 (9.77)	147 (7.61)	<0.001	147 (7.61)	147 (7.61)	1	
Tumor grade							
Well	16720 (9.75)	18 (0.93)		188(9.73)	18 (0.93)		
Moderately	102945 (60.02)	94 (4.87)		1132 (58.59)	94 (4.87)		
Poorly	22666 (13.21)	1254 (64.91)		261 (13.51)	1254 (64.91)		
Undifferentiated	4388 (2.56)	267 (13.82)		53 (2.74)	267 (13.82)		
Unknown	24809 (14.46)	299 (15.48)	<0.001	298 (15.42)	299 (15.48)	<0.001	
AJCC							
0, I, II	89273 (52.05)	416 (21.53)		996 (51.55)	416 (21.53)		
III, IV	75552 (44.05)	1469 (76.04)		862 (44.62)	1469 (76.04)		
Unknown	6703 (3.91)	47 (2.43)	<0.001	74 (3.83)	47 (2.43)	<0.001	
T stage							
Tis, T1, T2	59259 (34.55)	208 (10.77)		686 (35.51)	208 (10.77)		
T3, T4	97053 (56.58)	1521 (78.73)		1091 (56.47)	1521 (78.73)		
Unknown	15216 (8.87)	203 (10.51)	<0.001	155 (8.02)	203 (10.51)	<0.001	
N stage							
N0	104602 (60.98)	650 (33.64)		1173 (60.71)	650 (33.64)		
N1, N2	60351 (35.18)	1175 (60.82)		695 (35.97)	1175 (60.82)		
Unknown	6575 (3.83)	107 (5.54)	<0.001	64 (3.31)	107 (5.54)	<0.001	
M stage							
M0	138795 (80.92)	1176 (60.87)		1573 (81.42)	1176 (60.87)		
M1	32733 (19.08)	756 (39.13)	<0.001	359 (18.58)	756 (39.13)	<0.001	
Surgery							
No	26645 (15.53)	410(21.22)		291 (15.06)	410 (21.22)		
Yes	144883 (84.47)	1522 (78.78)	<0.001	1641 (84.94)	1522 (78.78)	<0.001	
Radiotherapy							
No	153802 (89.67)	1793 (92.81)		1744 (90.27)	1793 (92.81)		
Yes	17726 (10.33)	139 (7.19)	<0.001	188 (9.73)	139 (7.19)	0.006	
Chemotherapy							
No/Unknown	108671 (63.35)	873 (45.19)		1224 (63.35)	873 (45.19)		
Yes	62857 (36.65)	1059 (54.81)	<0.001	708 (36.65)	1059 (54.81)	<0.001	

In order to eliminate the impact of the difference in the number of patients with SRCC and NOS, we also conducted PSM to analyze these patient characteristics (Table 1). The characteristics between two groups were well balanced regarding sex, race, and age. Compared to NOS patients, SRCC patients presented higher metastatic rates (39.13% vs 18.58%) and higher treatment rates of chemotherapy (54.81% vs 36.65%). Results between SRCC and NOS patients were basically the same after PSM, and detailed clinicopathological characteristics are presented in Table 1.

### 3.2 Survival comparisons among metastatic or nonmetastatic SRCC and NOS patients

**Figure 2:**
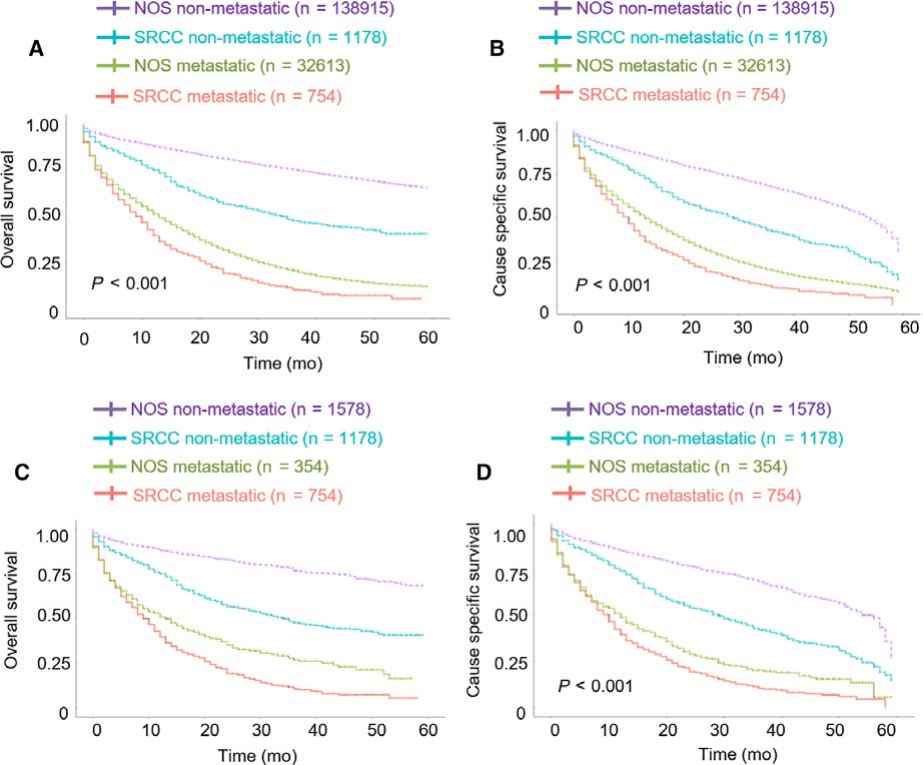
A, Overall survival among NOS and SRCC patients with and without metastasis. *P* < 0.001. B, Cause‐specific survival among NOS and SRCC patients with and without metastasis. *P* < 0.001. C, Overall survival among NOS and SRCC patients with and without metastasis after PSM *P* < 0.001. D, Cause‐specific survival among NOS and SRCC patients with and without metastasis after PSM *P* < 0.001. NOS, non‐SRCC colorectal cancer; SRCC, colorectal signet ring cell carcinoma; PSM, propensity score matching

With the aim of comparing survival differences among SRCC and NOS patients who had or did not have metastasis, we analyzed the survival curves and divided patients into four groups (Figure 2). Metastatic CRC patients had a worse survival than nonmetastatic CRC patients in both OS and CSS (*P* < 0.001). In addition, within the metastatic CRC patients, patients with metastatic SRCC had poorer survival than patients with metastatic NOS (*P* < 0.001). This implied that M1 stage and SRCC pathology were negative prognostic factors. The results were the same after PSM (in Figure 2C; *P* < 0.001 and Figure 2D; *P* < 0.001), when adjusted for age, sex, and race.

### 3.3 Occurrence frequency and prognostic factors among SRCC patients with site‐specific metastasis

In order to further explore the differences of SRCC patients with site‐specific metastasis, we analyzed six possible metastatic sites, including peritoneum metastasis, distant lymph node metastasis, liver metastasis, bone metastasis, lung metastasis, and brain metastasis. Occurrence frequencies of these metastatic sites were analyzed as shown in Table 2. Of note, we included one patient into a certain metastatic group as long as this patient was diagnosed with this specific metastatic site. Peritoneum metastasis and distant lymph node metastasis of SRCC patients were significantly more prevalent than that in NOS patients (17.65% vs 5.54%, *P* < 0.001; 11.08% vs 3.17%, *P* < 0.001, respectively). However, liver metastasis was less frequent in SRCC patients compared to that in NOS patients (6.88% vs 13.85%, *P* < 0.001). In all, Table 2 indicated that the peritoneum was the most frequent metastatic site in SRCC patients, but liver metastasis was not common in SRCC compared to non‐SRCC colorectal cancer patients.

**Table 2:** Patterns of distant metastases for NOS and SRCC patients with colorectal cancer

Variable	NOS n = 171 528 (%)	SRCC n = 1932 (%)	*P*
Peritoneum			
Yes	9507 (5.54)	341 (17.65)	
No	162021 (94.46)	1591 (82.35)	<0.001
Distant lymph node			
Yes	5444 (3.17)	214 (11.08)	
No	166084 (96.83)	1718 (88.92)	<0.001
Bone			
Yes	1839 (1.07)	59 (3.05)	
No	169689 (98.93)	1873 (96.95)	<0.001
Brain			
Yes	420 (0.24)	10 (0.52)	
No	171108 (99.76)	1922 (99.48)	0.030
Liver			
Yes	23755 (13.85)	133 (6.88)	
No	147773 (86.15)	1799 (93.12)	<0.001
Lung			
Yes	7802 (4.55)	54 (2.8)	
No	163726 (95.45)	1878 (97.2)	<0.001

Next, to further explore the factors that may influence long‐term survival of patient with SRCC/NOS, univariate and multivariate Cox regression analyses were performed to determine the independent prognostic factors (Table 3). The results showed that in univariate COX regression, age greater than 65 years (HR, 1.34, *P* < 0.001) and peritoneum metastasis (HR, 1.27, *P* = 0.042) were independent poor prognostic factors, while female sex (HR, 0.86, *P* = 0.048), T3/T3 stage (HR, 0.61, *P* = 0.003), surgery (HR, 0.62, *P* < 0.001), radiotherapy (HR, 0.56, *P* = 0.031), and chemotherapy (HR, 0.45, *P* < 0.001) were independent adverse prognostic factors. In multivariate COX regression, age greater than 65 years (HR, 1.22, *P* = 0.033) was an independent poor prognostic factor, while surgery (HR, 0.71, *P* = 0.003) and chemotherapy (HR, 0.45, *P* < 0.001) were independent adverse prognostic factors. However, peritoneum metastasis was not an independent prognostic factor in multivariate COX regression (*P* = 0.141), which may be for the limited SRCC patients who were diagnosed with specific metastatic sites.

**Table 3:** Univariate and multivariate analyses for SRCC patients with distant metastasis

Variable	Univariable			Multivariable		
	HR	95% CI	*P *value	HR	95% CI	*P *value
Age (y)						
<65	Ref			Ref		
≥65	1.34	1.15‐1.56	<0.001	1.22	1.02‐1.45	0.033
Sex						
Male	Ref			Ref		
Female	0.86	0.74‐1	0.048	0.99	0.83‐1.18	0.898
Race						
White	Ref					
Black	1.02	0.74‐1.41	0.905			
Other/Unknown	1	0.74‐1.36	0.986			
Tumor grade						
Well	Ref					
Moderately	1.51	0.39‐5.77	0.548			
Poorly	1.04	0.37‐2.97	0.935			
Undifferentiated	0.66	0.36‐1.23	0.191			
T stage						
Tis, T1, T2	Ref			Ref		
T3, T4	0.61	0.44‐0.84	0.003	0.69	0.48‐1.01	0.055
N stage						
N0	Ref					
N1, N2	0.85	0.71‐1.01	0.061			
Distant metastasis						
Liver	Ref			Ref		
Distant lymph node	1.07	0.8‐1.43	0.648	0.96	0.66‐1.38	0.814
Peritoneum	1.27	1.01‐1.6	0.042	1.23	0.93‐1.63	0.141
Surgery						
No	Ref			Ref		
Yes	0.62	0.53‐0.72	<0.001	0.71	0.57‐0.89	0.003
Radiotherapy						
No	Ref			Ref		
Yes	0.56	0.33‐0.95	0.031	0.81	0.45‐1.45	0.48
Chemotherapy						
No/Unknown	Ref			Ref		
Yes	0.45	0.39‐0.53	<0.001	0.45	0.37‐0.54	<0.001

### 3.4 Survival comparisons among SRCC patients with different metastatic sites

After analyzing the occurrence frequency and prognosis of site‐specific metastasis of SRCC, we next made a further analysis of the survival regarding different metastatic sites.

**Figure 3:**
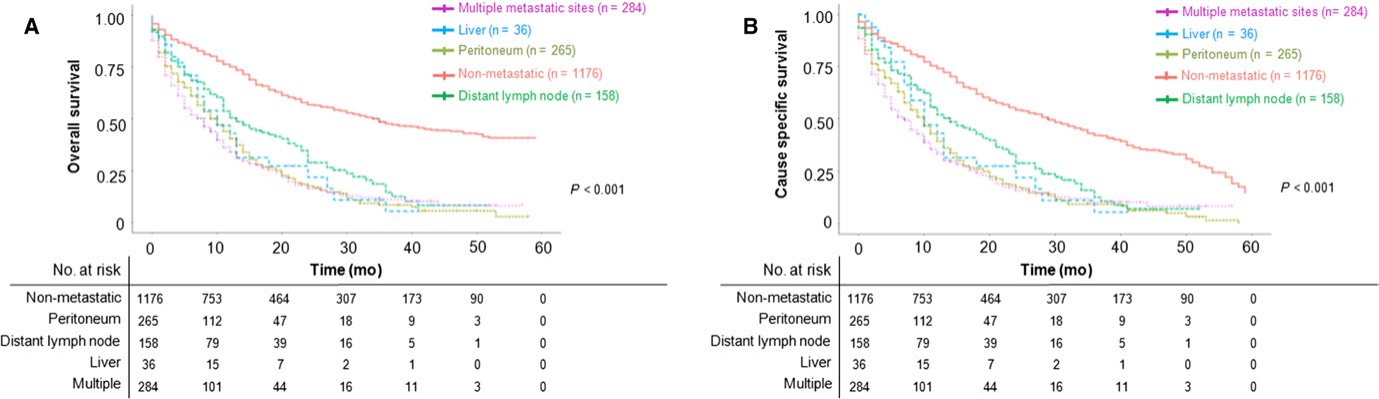
A, Overall survival among SRCC patients with distant metastatic sites (multiple, liver, peritoneum, distant lymph node, and nonmetastatic); *P* < 0.001. B, Cause‐specific survival among SRCC patients with distant metastatic sites (multiple, liver, peritoneum, distant lymph node, and nonmetastatic); *P* < 0.001. SRCC, colorectal signet ring cell carcinoma

Five‐year overall survival and cause‐specific survival among SRCC patients with different metastatic subtypes (1176 nonmetastatic, 265 peritoneum metastasis only, 158 distant lymph node metastasis only, 36 liver metastasis only, and 284 multiple metastatic sites) are presented in Figure 3. Lung, brain, and bone metastasis were not included in the subsequent analyses due to the limited patient number (6 bone metastasis only, 1 brain metastasis only, and 6 lung metastasis only). Five‐year OS and CSS among SRCC patients with all metastatic subtypes are in Supplementary Figure S1. The results of OS and CSS both suggested that compared to median survival time, survival of SRCC patients with multiple metastatic sites was the worst (OS = 7 months, CSS = 7 months, *P* < 0.001). Among single‐site metastasis of SRCC patients, SRCC patients with peritoneum metastasis had the worst survival, while patients with distant lymph node metastasis had the best relative survival (median OS: peritoneum metastasis = 9 months, distant lymph node metastasis = 13 months, liver metastasis = 10 months, *P* < 0.001; median CSS: peritoneum metastasis = 10 months, distant lymph node metastasis = 14 months, liver metastasis = 10 months, *P* < 0.001.). Thus, with these single metastatic sites, peritoneum was both the site with the highest frequency of occurrence and the site with the worst prognosis.

### 3.5 Comparisons of treatment strategies among SRCC patients with different metastatic sites

Because we compared the survival and occurrence frequency of SRCC patients with different metastatic sites, it was also important to identify improved treatment methods related to these patients.

**Figure 4:**
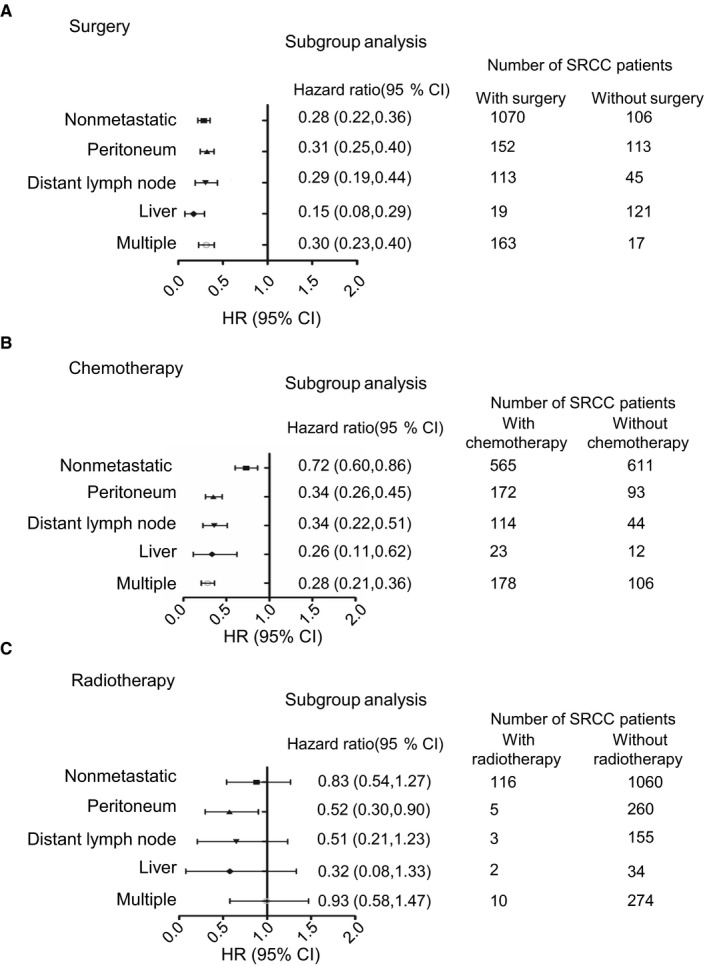
A, Subgroup analysis of surgery effect among SRCC patients with distant metastatic sites (multiple, liver, peritoneum, distant lymph node, and nonmetastatic); *P* < 0.001. B, Subgroup analysis of chemotherapy effect among SRCC patients with distant metastatic sites (multiple, liver, peritoneum, distant lymph node, and nonmetastatic); *P* < 0.001. C, Subgroup analysis of radiotherapy effect among SRCC patients with distant metastatic sites (multiple, liver, peritoneum, distant lymph node, and nonmetastatic); *P* > 0.001. SRCC, colorectal signet ring cell carcinoma; CI, confidence interval

Treatment strategies including surgery, radiotherapy, and chemotherapy were analyzed against metastatic SRCC patients as shown in Figure 4. For treatment effect of surgery, the outcomes indicated that survival benefits could be found both in nonmetastatic (HR = 0.28, *P* < 0.001) and metastatic SRCC patients with surgery performed (peritoneum, HR = 0.31; distant lymph node, HR = 0.29; liver, HR = 0.15; multiple, HR = 0.30; *P* < 0.001). However, among metastatic SRCC patients, there was little prognostic difference regarding the different metastatic sites. Survival after surgery as treatment was the best in nonmetastatic SRCC (HR = 0.28), while it was the worst in patients with peritoneum metastasis (HR = 0.31). Among these metastatic SRCC patients, the treatment effect of chemotherapy was much better in the metastatic groups (peritoneum, HR = 0.34; distant lymph node, HR = 0.34; liver, HR = 0.26; multiple, HR = 0.28; *P* < 0.001) compared to the nonmetastatic group (HR = 0.72, *P* < 0.001). Analysis of radiotherapy was not of statistical significance (*P* > 0.05) because of the few numbers of patients receiving radiotherapy.

Considering the significance of peritoneum metastasis, which was both the site with the highest occurrence frequency and the site with the worst prognosis, we further explored the impact of various combinations of treatments in SRCC patients with peritoneum metastasis only (Table 4). Patients who received radiotherapy only, surgery and radiotherapy, or chemotherapy and radiotherapy were not included due to the low patient numbers. The results implied that peritoneum‐metastatic SRCC patients who received chemotherapy only (HR = 0.33, *P* < 0.001) had a better survival than those who had surgery only (HR = 0.6, *P* = 0.028). Additionally, patients who received surgery and chemotherapy also had promising survival (HR = 0.19, *P* < 0.001), while patients who received surgery, chemotherapy, and radiotherapy all had the best survival (HR = 0.11, *P* = 0.003).

**Table 4:** Univariate analyses for SRCC patients with peritoneum metastasis

Variable	Univariable		
	HR	95% CI	*P *value
Therapy			
No treatment	Ref		
Surgery only	0.6	0.39‐0.95	0.028
Chemotherapy only	0.33	0.21‐0.52	<0.001
Surgery and chemotherapy	0.19	0.13‐0.3	<0.001
Surgery and chemotherapy and radiotherapy	0.11	0.03‐0.48	0.003

Collectively, these results suggested that chemotherapy should be the first consideration for treatment of metastatic colorectal SRCC. Also, combined treatment of chemotherapy and surgery led to increased survival for colorectal SRCC patients with distant metastasis, especially those with peritoneum metastasis.

## 4 DISCUSSION

Signet ring cell carcinoma (SRCC) has been associated with poor prognosis and is defined as one of the most malignant cancers compared with other colorectal cancers.^7^ Most frequently, signet ring cells are found to be present in colon, rectum, stomach, prostate, and bladder, with a large proportion of multiple metastasis sites. Signet ring cells present round shapes and have abundant mucins in the cytoplasm.^6^ Previously, retrospective studies have analyzed the prognostic factors of primary colorectal SRCC,^5^, ^14^, ^15^ but failed to compare the differences of survival and prognosis related to specific metastatic sites.

In this large population‐based study, we analyzed 173 460 CRC patients from 2010 to 2014 from the SEER database, including 1932 patients diagnosed with colorectal SRCC, which was similar to the numbers reported in previous studies consisting of all stages of CRC patients.^17^, ^18^ Among this cohort, our study found that SRCC presented unique characteristics of low differentiation, high tumor grade, and high T/M/N stage, and this finding was consistent with results derived from other databases.^17^ Our study and other systematic investigations together showed that female sex and younger age were protective prognostic factors of colorectal SRCC.^19^, ^20^ Additionally, it was reported that signet ring cells lack the ability to maintain cell to cell contact through ErbB2/ErbB3 pathway actions, and secretion of Muc4 and, therefore, diffusely infiltrate the stroma to form invasion and metastasis.^21^ This mechanism may partly explain the high metastasis rate and poor prognosis of colorectal SRCC compared to non‐SRCC colorectal cancers, as derived from our analyses. The relationship between molecular factors and the metastatic mechanism of SRCC should be further investigated in the future.

In our analysis, the results showed that SRCC patients with peritoneum metastasis had the largest incidence among site‐specific SRCC metastasis and had the poorest survival. This finding was consistent with research conducted by Van Oudheusden et al,^22^ which suggested SRCC patients more often developed peritoneal and ovarian metastases. The less occurrence of operations for curative surgery also reduced the survival of SRCC patients with peritoneal metastasis. This also suggested that more attention needs to be paid to the future treatment of SRCC patients with peritoneal metastases. According to our results, distant lymph node metastasis was the second highest site in SRCC metastasis, which may explain the results in previous studies that distant lymph node metastasis was a significant independent prognostic indicator in SRCC patients.^23^ Another important finding in our study was that, compared to colorectal non‐SRCC, SRCC patients presented with a lower rate of liver metastasis, which has not been reported before, and may partly be due to the easier peritoneal metastasis approach.

In agreement with the results regarding treatment methods targeting site‐specific metastasis of colorectal SRCC patients in our study, our additional main findings were as follows: (a) There was no large difference in prognosis of nonmetastatic and metastatic SRCC patients with surgery performed; (b) metastatic SRCC patients with chemotherapy had better survival than nonmetastatic SRCC patients who received chemotherapy; and (c) for colorectal SRCC patients with peritoneal metastases, chemotherapy was the first choice of treatment, while combined treatment of surgery, chemotherapy, and radiotherapy provided the best survival outcome. The role of surgery in our analysis agreed with Fu et al,^14^ who claimed that rare surgical value was noted in SRCC of resectable metastatic colorectal cancer. However, the impact of chemotherapy was the opposite from several previous studies. Lee et al and Pande et al found that SRCCs were less sensitive to commonly used chemotherapy drugs like 5‐FU, oxaliplatin, and irinotecan, resulting in limited treatment outcomes.^24^, ^25^ Interestingly, in 2015, a large population‐based study with 1972 colorectal SRCC patients, by Hugen et al^5^ suggested a comparable prognosis benefit from adjuvant chemotherapy in SRCC patients. There are some possible reasons for the contradictory results. For example, we did not consider the impact of preoperative or postoperative adjuvant therapy in our study, and the specific chemotherapy regimen was unknown. Thus, we strongly recommend future studies to reevaluate the effect of chemotherapy on patients with metastatic colorectal SRCC, in a larger population. Lastly, due to the limited patient number of SRCC patients who received radiotherapy, we could not evaluate the impact of radiotherapy on nonmetastatic or metastatic colorectal SRCC and only a few studies assessing the efficacy of radiotherapy alone or combined with chemotherapy in SRCC patients have been reported.^9^, ^26^ Therefore, we also recommend further investigation on the role of radiotherapy in SRCC.

Our study had several strengths. We assessed a large number of patients from a national population‐based data in the United States, and our study avoided the biases associated with single institution records or limited sample sizes. Also, we employed PSM to exclude the interference of results caused by the difference in patient numbers. However, our present study also had some potential limitations. First, this SEER‐based research was a retrospective study and inaccuracy inevitably existed in our data analysis process. Second, the poor diagnosis of early‐stage SRCC in colon and rectum and the limited number of radiotherapy‐receiving patients could have affected the number of SRCC patients in our study, which was associated with the derived conclusions. Third, the data of targeted therapies and immune therapies toward colorectal SRCC were not included in the SEER database, which may be new treatment approaches for colorectal SRCC patients.

## 5 CONCLUSIONS

Our study showed that colorectal SRCC was a distinct entity with different biological behavior, pathological features, and treatment responses compared to non‐SRCC colorectal cancers. The peritoneum was both the site with the highest metastatic frequency and the site with the worst prognosis in colorectal SRCC. Also, we recommend chemotherapy as the first consideration in metastatic colorectal SRCC treatment. In the future, more studies are needed to further evaluate treatments toward site‐specific SRCC and elucidate colorectal SRCC patient selection criterion for surgery, chemotherapy, and radiotherapy.

## CONFLICT OF INTEREST

The authors declare no conflict of interest.
