# Supplementary figures and images for: Chemotherapy is associated with increased survival from colorectal signet ring cell carcinoma with distant metastasis: A Surveillance, Epidemiology, and End Results database analysis
Source: Cancer Med. 2019 Mar 12;8(4):1930–40. doi: 10.1002/cam4.2054 (PMC6488115; doi:10.1002/cam4.2054)

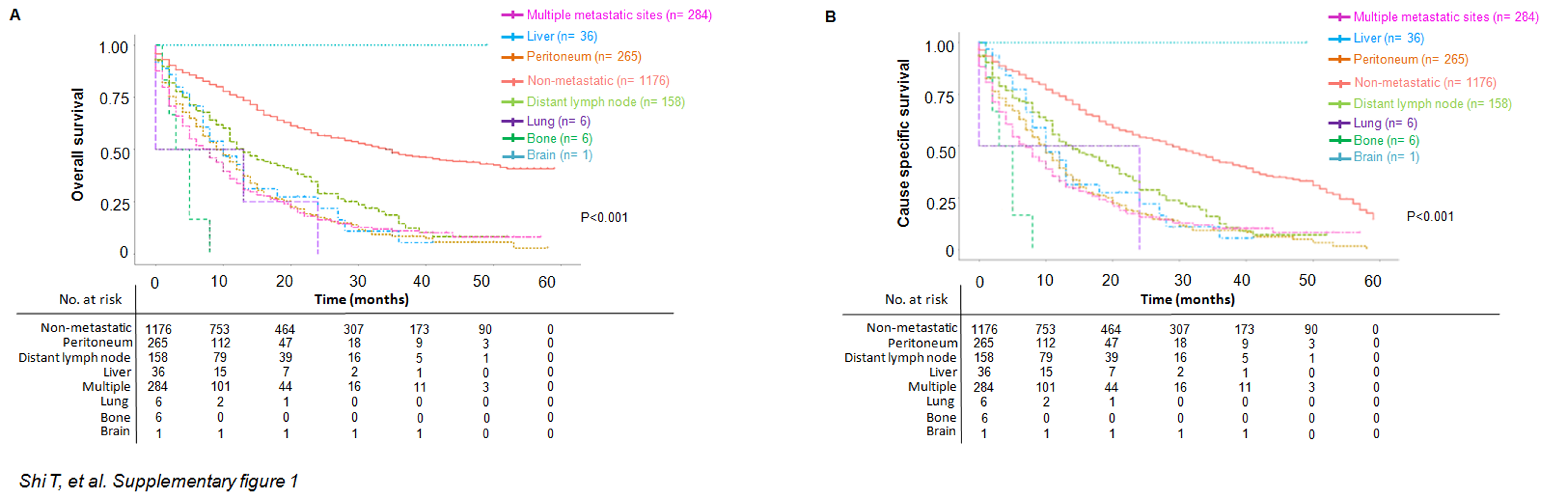

Supplement: Supplementary file 1 [file CAM4-8-1930-s001.tif]
